# Reinforcement of perceptual inference: reward and punishment alter conscious visual perception during binocular rivalry

**DOI:** 10.3389/fpsyg.2014.01377

**Published:** 2014-12-03

**Authors:** Gregor Wilbertz, Joanne van Slooten, Philipp Sterzer

**Affiliations:** Visual Perception Lab, Department of Psychiatry and Psychotherapy, Charité – Universitätsmedizin BerlinBerlin, Germany

**Keywords:** binocular rivalry, reward, punishment, perceptual inference, visual perception

## Abstract

Perception is an inferential process, which becomes immediately evident when sensory information is conflicting or ambiguous and thus allows for more than one perceptual interpretation. Thinking the idea of perception as inference through to the end results in a blurring of boundaries between perception and action selection, as perceptual inference implies the construction of a percept as an active process. Here we therefore wondered whether perception shares a key characteristic of action selection, namely that it is shaped by reinforcement learning. In two behavioral experiments, we used binocular rivalry to examine whether perceptual inference can be influenced by the association of perceptual outcomes with reward or punishment, respectively, in analogy to instrumental conditioning. Binocular rivalry was evoked by two orthogonal grating stimuli presented to the two eyes, resulting in perceptual alternations between the two gratings. Perception was tracked indirectly and objectively through a target detection task, which allowed us to preclude potential reporting biases. Monetary reward or punishments were given repeatedly during perception of only one of the two rivaling stimuli. We found an increase in dominance durations for the percept associated with reward, relative to the non-rewarded percept. In contrast, punishment led to an increase of the non-punished compared to a relative decrease of the punished percept. Our results show that perception shares key characteristics with action selection, in that it is influenced by reward and punishment in opposite directions, thus narrowing the gap between the conceptually separated domains of perception and action selection. We conclude that perceptual inference is an adaptive process that is shaped by its consequences.

## INTRODUCTION

We perceive the world through our senses, but the sensory input that reaches our brains is a fundamentally impoverished source of information about the external world. Our brains must go beyond what is directly available in the sensory data through a process of interpretation, or inference, to achieve the rich percepts of conscious awareness. Accordingly, current theories of brain function consider perception an active inference process, in which the contents of conscious perception are generated by neural computations that rest on predictive models of how the sensory data are caused (Friston, 2005). Thinking the notion of perception as an active inference process through to the end inevitably leads to a blurring of boundaries between the conceptually separated domains of perception and action, because perceptual inference implies the construction of perceptual experience as an active process. Consequently, it is plausible to assume that this active perceptual process is shaped according to the same principles as actions in the strict sense. A key characteristic of actions is that they are modulated by learning through reinforcement and punishment, which is known as instrumental conditioning (Skinner, 1938). According to the ‘law of effect’ (Thorndike, 1911), actions leading to favorable outcomes are more likely to be selected again in the future, while aversive consequences lower the probability of an action. Here we reasoned that this law might not only apply to actions, but also to the active process of perceptual inference. In other words, association of a perceptual experience with favorable consequences should increase the probability of this particular outcome of perceptual inference in the future, while aversive consequences should conversely decrease this probability.

Previous studies have demonstrated a modulation of perceptual inference by value (Proshansky and Murphy, 1942; Schafer and Murphy, 1943; Haggard and Rose, 1944; Alpers et al., 2005; Balcetis and Dunning, 2006; Fleming et al., 2010; Summerfield and Koechlin, 2010; Anderson et al., 2011c; Balcetis et al., 2012; see also Riccio et al., 2013, for review). For example, ambiguous face-house images (overlaid pictures that could be perceived as either a house or a face) were more often categorized as houses when pay-off matrices suggested this perceptual decision to be advantageous (Fleming et al., 2010). This is in line with a number of other findings that suggest that uncertainty with regard to a percept (e.g., due to ambiguous or noisy stimuli), is resolved in accordance not only with expectations (Schmack et al., 2013), but also current needs (Sanford, 1936; Balcetis and Dunning, 2010; Radel and Clement-Guillotin, 2012), and emotional states (Stefanucci et al., 2008; Anderson et al., 2011b; Sterzer et al., 2011). However, most of these studies relied on participants’ report of what they see and methodological concerns have repeatedly been raised (Erdelyi, 1974; Firestone and Scholl, 2014). Obviously, in the context of motivational and emotional factors, the participants’ reports of what they perceive are likely to be influenced by what they wish to perceive in order to obtain a favorable outcome (cf. reward response bias, Rees and Fishbein, 1970) as well as by what they think the experimenter wishes them to see (cf. social factors in experiments, Orne, 1962). A key challenge when investigating the effect of reinforcement on perception is thus to preclude confounding effects from such biases. We therefore devised an experimental paradigm in which subjective perceptual experience was indirectly inferred from an orthogonal target detection task (similar to Yu and Blake, 1992; Nguyen et al., 2001; Chong et al., 2005; Alpers and Gerdes, 2007; Balcetis et al., 2012). This procedure provided us with an objective measure of the participants’ subjective perceptual experience, thus precluding any voluntary reporting bias.

The aim of the present study was to test the hypothesis that perceptual inference is shaped by instrumental learning according to the same principles as action selection. The inferential nature of perception is immediately evident in situations where sensory information is ambiguous or conflicting. A typical example is binocular rivalry, which is evoked by the presentation of incompatible images to the two eyes, resulting in spontaneous alternation between the two possible outcomes of inference and thus perception of either one or the other image at a time (Blake and Logothetis, 2002; Tong et al., 2006). Here we used binocular rivalry to probe the effect of instrumental conditioning on perceptual inference. In two behavioral experiments, we coupled perception of only one of two rivaling grating stimuli with monetary reward or punishment, respectively.

According to our hypothesis that perceptual inference is shaped by instrumental learning, we expected that reward should bias inference toward the associated perceptual outcome, leading to increased dominance durations for the rewarded percept during binocular rivalry. Conversely, we expected decreased dominance durations for the perceptual outcome associated with punishment. Importantly, the punishment condition was critical for distinguishing the effects of instrumental conditioning from those of affective stimulus salience. Previous research suggests that the affective salience of a stimulus facilitates its perception under conditions of binocular rivalry, both for stimuli with positive and negative valence (Alpers et al., 2005; Alpers and Gerdes, 2007; Stein and Sterzer, 2012). If coupling of a perceptual outcome with punishment enhanced the salience of the respective grating stimulus, one would expect *increased* dominance durations for this stimulus, as previously reported for effects of classical fear conditioning (Alpers et al., 2005; Anderson et al., 2011c). In contrast, if perceptual inference is shaped by instrumental conditioning, one would expect avoidance of the punished perceptual outcome and thus *decreased* dominance durations, in analogy to instrumental conditioning of decision or action selection.

## MATERIALS AND METHODS

### PARTICIPANTS

Thirty-seven participants completed Experiment 1, 34 completed Experiment 2. All volunteers gave written informed consent to participation in this study, which was approved by the local ethics committee of Charité – Universitätsmedizin Berlin. Participants were right-handed and mostly medical students (Experiment 1: 11 males, 26 females, Experiment 2: 15 males, 19 females), aged 18–32 (*M* = 23.68, SD = 2.92) and 18–35 (*M* = 23.09, SD = 3.74) in Experiments 1 and 2, respectively. Participants had no mental or neurological disorders and had normal or corrected-to-normal vision (the only tolerated exception was corrected-to-normal vision through contact lenses; none of the participants suffered from strabismus). They were naïve with regard to binocular rivalry and had never participated in any vision experiment before.

### MATERIAL

**FIGURE 1:**
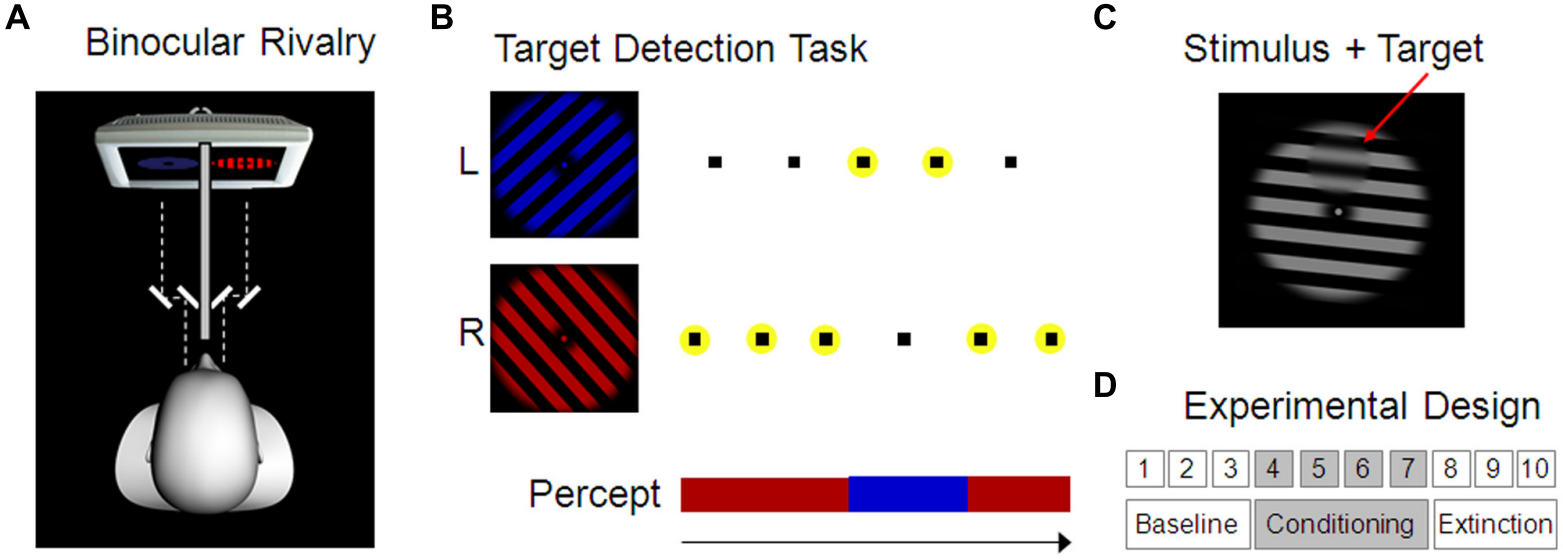
**Experimental design. (A)** Binocular rivalry setup. Red and blue grating stimuli were presented to the left and right eye separately using a mirror stereoscope. As a consequence, conscious visual perception alternated spontaneously between the two stimuli. **(B)** Target stimuli (here indicated as small black squares) were presented alternatingly superimposed on the two grating stimuli in four possible positions and had to be reported by the participant using key presses. Targets on the currently dominant stimulus were clearly visible whereas those on the currently suppressed stimulus were invisible. Correct responses to a target, i.e., correct identification of the target position (here indicated by a yellow circle), was therefore used to indirectly assess the current conscious perception of the participant. **(C)** Example of a target, i.e., a small smoothed disk upon the grating stimulus (here depicted in the top position). **(D)** Experimental Design comprising 10 blocks with 3 min each, separated in baseline, conditioning, and extinction blocks. During the conditioning blocks, one of the two percepts was rewarded (Experiment 1) or punished (Experiment 2) by monetary gains and losses, respectively.

Participants sat in a quiet and dark room in front of a computer screen (60 Hz). They watched stimuli through a mirror stereoscope (see **Figure 1A**). Two red or blue rotating grating stimuli, adapted from Haynes and Rees (2005), were used in a binocular rivalry paradigm, i.e., one stimulus was presented to the right eye and one to the left (color and eye were counterbalanced across participants). As a consequence, conscious perception alternated continuously between the two stimuli. Each stimulus consisted of an annulus-shaped square-wave grating (1.0° visual angle width of stripes, 1.3 and 11.6° visual angle diameter of inner and outer circle, respectively), spatially smoothed in front of a black background (0.74 cd/m^2^). Grating stimuli were oriented orthogonally and rotated with 360° per second around a central dot (0.5° visual angle diameter, colored red or blue, respectively). The brightness of the red stimulus was fixed at 10.2 cd/m^2^, whereas the blue stimulus was individually adjusted to match the brightness of the red stimulus (on average 6.7 cd/m^2^, range 4.7–8.0 cd/m^2^; see *Procedure* below).

Targets were small circular areas (3.7° visual angle diameter) within which the grating was spatially smoothed (see **Figure 1C**). Presentation of these targets was faded in and out over 700 ms. Target stimuli did not rotate like the grating stimuli but remained stationary. Two parameters that had an effect on the visibility of the target were individually adjusted for each participant in order to achieve optimal performance on the target detection task (smoothness of the target border as defined by the border width: *M* = 0.3° visual angle, range 0.2–0.5; and degree of smoothing of the background grating within the target area as defined by the FWHM of a Gaussian Filter: *M* = 2.8° visual angle, range 2.5–3.0). Target stimuli appeared randomly at four possible positions (up, down, left, and right) with an inter-stimulus-interval of 675 ms (±200 ms jitter) and had to be reported by pressing one of four keys. In line with a previous report of a strong reduction in contrast sensitivity on the currently non-dominant eye (Baker and Cass, 2013), it was possible to render targets effectively invisible on the suppressed eye but still clearly visible on the dominant eye (Chong et al., 2005). Using an alternating presentation scheme of targets on the left- and right-eye stimulus then allowed us to track dominance and suppression of the rivaling gratings indirectly based on correctly detected and missed targets, respectively (see **Figure 1B** and Figure S3 in the Supplementary Material for more details). Catch trials with targets presented simultaneously on both stimuli (at the same position) were randomly interspersed at every 35th target presentation on average.

### PROCEDURE

At the beginning participants were instructed about the general procedure and the task. In Experiment 1, they were informed about a starting balance of € 8, and that they could win money throughout the task up to a balance of € 20; in Experiment 2, they started at a balance of € 25 and were informed that they would lose money during the task. Without looking through the mirror stereoscope, participants were first shown the stimulus in gray as well as an example target upon it. Next, the mirror stereoscope was adjusted individually to ensure proper fusion of the dichoptically presented images. Importantly, participants were kept naïve with regard to dichoptic presentation by using only identical stimuli for left and right eye during mirror adjustment. After that, the colors of the rivaling stimuli were made equiluminant using heterochromatic flicker photometry (Rood, 1893). In brief, red and blue frames alternated while participants minimized subjectively perceived flickering by adjusting the luminance of the blue stimulus. Finally, before starting the main experiment, participants performed several blocks of training on the target detection task with the rivaling stimuli. They were instructed to report every target they detected by pressing one of the four arrow buttons on a computer keyboard (up, down, left, or right). Furthermore, it was stated that response time did not matter as long as the response appeared within a time window of 1 s. During training, target visibility was high in the beginning and was continuously reduced from block to block in order to meet the criteria of (a) no more than two consecutive targets missed, and (b) no more than two consecutive targets hit (note: consecutive targets appeared alternating on the left and right eye). With rule (a), we aimed to minimize the amount of time during which participants did not respond to any targets, neither those presented on the left nor those on the right eye (because no percept could be inferred for this period of time). With rule (b), we aimed to minimize the amount of time during which participants responded to targets presented on both eyes, i.e., targets on the left as well as the right eye (because, again, we would not be able to infer the percept based on such performance). Target visibility was adjusted manually by the experimenter (via adaptation of target area and border smoothness, see Material section above) after each of three or more training runs. Optimal adjustment of target visibility to meet these two criteria overall was achieved after 3.5–12.4 min of training (*M* = 6.2, SD = 1.8).

The main task consisted of 10 blocks of 3 min each, divided into three baseline, four conditioning, and three extinction blocks (see **Figure 1D**). Participants knew that no reward or punishment was delivered at the beginning of the task. Immediately before the fourth block, i.e., before the start of the first conditioning block, the following additional instruction was given: ‘From now on, you will sometimes hear the sound of a falling coin during one of the colors (red or blue) and this means that € 0.10 have been added to (Experiment 1)/subtracted from (Experiment 2) your balance. Your task is still to respond to every target you see, just as before.’ Reward/punishment was delivered every 2 s during the continuous percept of one stimulus with a probability of 50% (partial reinforcement schedule). Exact delivery time was determined on the basis of cumulative percept duration and was therefore not systematically related to percept onset or button press. During extinction blocks no reward/punishment was delivered any more.

At the end of the experiment, participants were reimbursed (according to their final balance) and answered a written question about possible strategies during the task. In a semi-structured verbal interview, participants were asked about their own hypotheses regarding the experiment, the origin of color changes (self-induced or physical changes on the computer screen), any association with reward/punishment delivery, possible associations between this delivery and color of the stimulus, as well as a 2-alternative forced choice (2AFC) question regarding color and reward/punishment (red or blue).

### ANALYSIS

Monetary gains (Experiment 1) and losses (Experiment 2) were delivered contingent on participants’ conscious perception. To that aim, conscious perception had to be inferred online (i.e., during the experiment) from performance in the target detection task (see also Figure S3 in the Supplementary Material for an illustration). Estimated perception was re-evaluated after each target based on the following rule: A stimulus is considered perceptually dominant if the last target presented on that stimulus was correctly detected (hit) and if the last and/or next target on the stimulus presented to the respective other eye was missed. Hits were defined as correct identification of a targets’ location via button press (up, down, left, or right) within a time window of 1 s after 50% of full target presentation (targets were faded in and out, see above), misses were defined as no response within this time window or wrong localization of the current target. Main outcome measures were median dominance durations based on the target detection performance. Effects of reward and punishment were tested comparing the two percepts during conditioning, corrected by the corresponding difference during baseline in paired *t*-tests:

(Re⁢w⁢a⁢r⁢d⁢e⁢dC⁢o⁢n⁢d⁢i⁢t⁢i⁢o⁢n⁢i⁢n⁢g−Re⁢w⁢a⁢r⁢d⁢e⁢dB⁢a⁢s⁢e⁢l⁢i⁢n⁢e⁢)⁢v⁢s⁢.(N⁢o⁢n⁢r⁢e⁢w⁢a⁢r⁢d⁢e⁢dC⁢o⁢n⁢d⁢i⁢t⁢i⁢o⁢n⁢i⁢n⁢g−N⁢o⁢n⁢r⁢e⁢w⁢a⁢r⁢d⁢e⁢dB⁢a⁢s⁢e⁢l⁢i⁢n⁢e)

$$\begin{array}{c} \left(\begin{array}{c} {\begin{array}{c} P\,u\,n\,i\,s\,h\,e\,d \end{array}}_{C\,o\,n\,d\,i\,t\,i\,o\,n\,i\,n\,g}-P\,u\,n\,i\,s\,h\,e\,d_{B\,a\,s\,e\,l\,i\,n\,e} \end{array}\right)\,v\,s\,.\\ \left(\begin{array}{c} {\begin{array}{c} N\,o\,n\,p\,u\,n\,i\,s\,h\,e\,d \end{array}}_{C\,o\,n\,d\,i\,t\,i\,o\,n\,i\,n\,g}-N\,o\,n\,p\,u\,n\,i\,s\,h\,e\,d_{B\,a\,s\,e\,l\,i\,n\,e} \end{array}\right) \end{array}$$

This was done in order to account for possible general changes in task performance (perceptual learning with regard to target detection, higher task motivation during conditioning) as well as potential baseline differences between the two percepts. Statistical tests were two-tailed, except for the main directed hypothesis regarding a reward effect in Experiment 1. Error bars in figures denote within-subject SEs (Cousineau, 2005).

In Experiment 1, we additionally performed one run with direct perceptual reports to validate our procedure of assessing dominance duration based on the target detection task. Before training, participants were asked to report red and blue percepts using two buttons during a 3-min presentation of the original binocular rivalry stimuli (red and blue on the left and right eye, respectively, counterbalanced across participants). Average percept durations based on this direct report were compared to average percept durations derived from the target detection performance during the next succeeding run after training (first baseline run) using Pearson correlation coefficient (note, since this correlation refers to different runs it cannot be expected to be perfect given random changes in binocular rivalry dynamics over time). In addition, distributions of percept durations from all blocks of Experiment 1 were compared to distributions of these directly reported percept durations using paired *t*-tests on the parameters of fitted gamma functions.

## RESULTS

**FIGURE 2:**
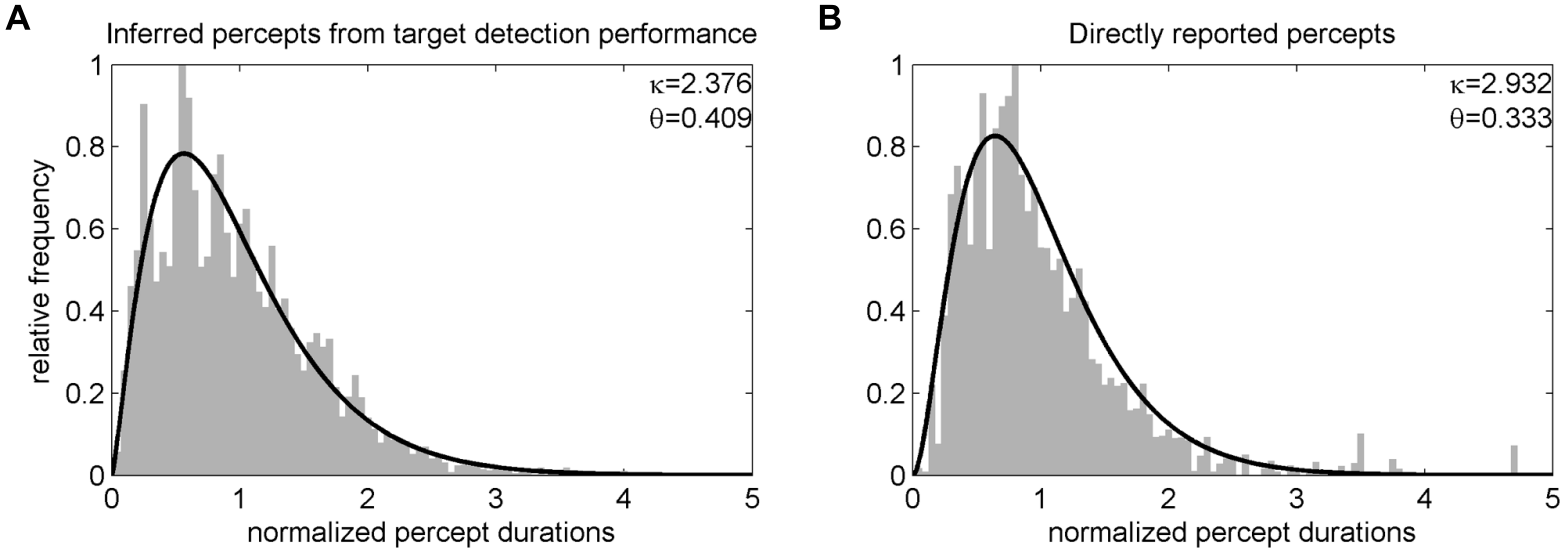
**Distribution of normalized percept durations of Experiment 1.** Before averaging across participants, individual percepts were normalized, i.e., divided by the corresponding participant’s mean phase duration (cf. Leopold and Logothetis, 1996). The resulting group level distributions of percept durations inferred from the target detection task **(A)** and directly reported **(B)** could both be well fitted by a gamma function (black line).

In Experiment 1, analysis of target detection performance revealed a hit rate of *M* = 92.56% (SD = 5.97) for catch trials (i.e., targets that could be detected independently of the current percept). Average percept durations determined by the target detection task were significantly correlated with percept durations derived from direct report of participants as verified in an independent experimental run [*r*(35) = 0.55, *p* < 0.001] but were generally longer (*M* = 6.08, SD = 3.37 s) than those directly reported [*M* = 4.63, SD = 1.93 s; *t*(36) = 2.91, *p* = 0.006]. Distributions of perceptual dominance durations were skewed and could be approximated by a non-symmetrical gamma function (see **Figure 2**). A comparison of the parameters of gamma functions fitted to each participant’s unnormalized percept durations showed no significant difference for the scale parameter 𝜃 [target detection task: *M* = 3.07, SD = 2.43; direct report: *M* = 2.20, SD = 4.06; *t*(36) = -1.16, *p* = 0.255, paired *t*-test] but a difference for the shape parameter κ [target detection task: *M* = 2.57, SD = 0.63; direct report: *M* = 3.92, SD = 1.55; *t*(36) = 5.79, *p* < 0.001].

**FIGURE 3:**
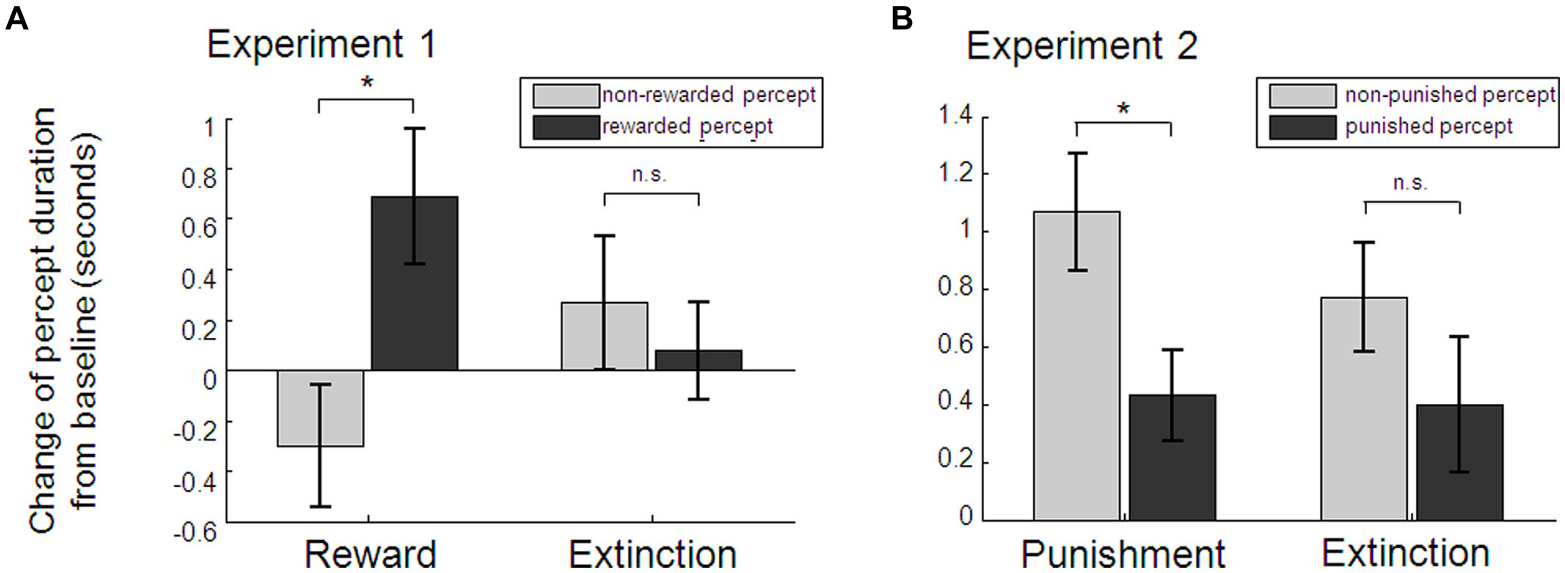
**Average percept durations as obtained by the target detection task.** Relative to baseline blocks, rewarded percepts were significantly longer than non-rewarded percepts during the conditioning blocks in Experiment 1 **(A)**. Punished percepts during the conditioning blocks in Experiment 2 were significantly shorter than non-punished percepts compared to baseline **(B)**. Reward and punishment effects were not persistent as indicated by no significant differences (n.s.) between percept durations during the extinction blocks. Note: sample sizes were *n* = 37 and *n* = 34 for Experiment 1 and 2, respectively; *p < 0.05, paired *t*-test. Error bars denote within-subject SEs.

Baseline percept durations did not differ between left- (*M* = 6.07, SD = 3.45) and right-eye [*M* = 6.23, SD = 3.09; *t*(36) = 0.39, *p* = 0.702], red (*M* = 6.29, SD = 2.93) and blue [*M* = 6.01, SD = 3.58; *t*(36) = 0.71, *p* = 0.483], or the later-on rewarded (*M* = 5.98, SD = 2.83) and later-on non-rewarded percept [*M* = 6.32, SD = 3.63; *t*(36) = 0.85, *p* = 0.399]. As hypothesized, baseline-corrected perceptual dominance durations of the rewarded percept during conditioning blocks were significantly longer than those of the non-rewarded percept [Student’s *t*-test *t*(36) = 2.18, *p* = 0.018, one-tailed, Cohen’s *d* = 0.36; see **Figure 3A**]. While there was a numerical change for the two percepts in opposite directions, neither the rewarded nor non-rewarded percept taken alone changed significantly from baseline to conditioning [*t*(36) = 1.46, *p* = 0.152, and *t*(36) = -1.27, *p* = 0.212, respectively]. During extinction, previously rewarded and non-rewarded percepts did not differ any more [*t*(36) = 0.47, *p* = 0.642]. The extinction-effect, i.e., the interaction between baseline-corrected rewarded and non-rewarded percept during conditioning and during extinction, was not significant [2 × 2 factorial ANOVA with the factors percept (rewarded vs. unrewarded) and experimental phase (conditioning vs. extinction): *F*(1,36) = 2.83, *p* = 0.101]. Eye movement recordings in a subset of 18 participants showed no evidence for a difference in the frequency of eye blinks in the two perceptual states (see Figure S1 in the Supplementary Material for more details). Moreover, detailed debriefing at the end of the experiment indicated that awareness of the aim of the study was generally low (65.5% of participants correctly guessed the rewarded color in the 2AFC question which is marginally above chance according to the binomial test: *p* = 0.068; see Supplementary Material for detailed results of the debriefing). However, in order to preclude any related confounding factors that might have driven the observed effect of reward on perception, we performed a control analysis excluding participants who showed any signs of possible confounding factors: (a) awareness of the subjective nature of color changes (i.e., notification of the effect of blinks on color change probability), (b) reports of any strategy regarding one stimulus or one color only (e.g., temporarily stop pressing for targets on one stimulus), (c) many missed targets, resulting in overall less than 70% of time where a percept could be inferred based on the target performance, (d) strong eye asymmetry or color preference, which resulted in significantly different percept durations during the baseline blocks (*p* < 0.05 of the Mann–Whitney *U* test for percept durations of the one vs. the other stimulus). 22 participants met at least one of these criteria (*n* = 5, 1, 12, 9 met a, b, c, d, respectively). Analysis of the remaining 15 participants revealed identical results, with a significant effect of reward on median dominance duration [*t*(14) = 1.85, *p* = 0.043, one-tailed, Cohen’s *d* = 0.48; see Figure S2 in the Supplementary Material]. Again there was no prolonged effect during extinction blocks [*t*(14) = -0.47, *p* = 0.648], no significant extinction effect [*F*(1,14) = 1.58, *p* = 0.230], and neither the rewarded nor non-rewarded percept taken alone changed significantly from baseline to conditioning [*t*(14) = 0.78, *p* = 0.446, *t*(14) = -1.61, *p* = 0.129, respectively].

In Experiment 2, a new group of participants completed the same task as in Experiment 1, with the only exception that one of the two perceptual states was now coupled with monetary loss instead of gain. Targets in catch trials were correctly detected in *M* = 93.11% (SD = 7.73). Baseline perceptual durations determined by the target detection task indicated no significant differences comparing left (*M* = 4.74, SD = 1.78) and right-eye [*M* = 4.99, SD = 1.76; *t*(33) = 0.99, *p* = 0.330], later-on punished (*M* = 4.98, SD = 1.83) and later-on non-punished [*M* = 4.75, SD = 1.71; *t*(33) = -0.88, *p* = 0.384] but a significantly longer duration of the red (*M* = 5.30, SD = 1.61) as the blue percept [*M* = 4.43, SD = 1.81; *t*(33) = 4.09, *p* < 0.001]. Baseline-corrected dominance durations of the percept that was paired with monetary loss were significantly shorter than non-punished percepts during conditioning blocks [*t*(33) = 2.77, *p* = 0.009, two-tailed, Cohen’s *d* = 0.47; see **Figure 3B**]. Interestingly, this was due to a significant increase of the non-punished percept [*t*(33) = 3.77, *p* = 0.001], whereas the punished percept did not change significantly [*t*(33) = 1.47, *p* = 0.152]. As in the first Experiment, baseline-corrected percept durations did not differ any longer during extinction [*t*(33) = 1.15, *p* = 0.259] and there was no significant extinction effect [*F*(1,33) = 0.83, *p* = 0.369]. Again, participants were relatively unaware of the aim of the study (67.7% correctly guessed the punished color in a 2AFC question, *p* = 0.023; see Supplementary Material for detailed results of the debriefing). To rule out possible effects of binocular rivalry awareness, voluntary strategies, task performance or baseline asymmetries, we again applied the same criteria as in Experiment 1 (*n* = 6, 4, 12, 9 met criteria a, b, c, d, respectively) to define a conservative subsample (*n* = 15), which yielded identical results [difference between punished and non-punished percept *t*(14) = 2.29, *p* = 0.038, two-tailed; Cohen’s *d* = 0.59; increase of the non-punished percept between baseline and punishment blocks *t*(14) = 3.03, *p* = 0.009, but no difference of the punished percept *t*(14) = 1.58, *p* = 0.136; no prolonged punishment effect during extinction blocks *t*(14) = 0.22, *p* = 0.828; no extinction effect *F*(1,14) = 1,54, *p* = 0.235; see Supplementary Material for further details].

Combined analysis of data from both experiments revealed that the increased duration of rewarded percepts in Experiment 1 and the decreased duration of punished percepts in Experiment 2 resulted in a significant 2-way interaction effect between type of conditioning (reward vs. punishment) and percept (conditioned vs. non-conditioned, *F*(1,69) = 9.66, *p* = 0.003 for the whole samples, and *F*(1,28) = 7.35, *p* = 0.011 for the conservative subsamples). Thus, reward and punishment had opposite effects on perceptual dominance durations.

## DISCUSSION

The results of our two experiments show that perceptual inference during binocular rivalry is shaped by its consequences, in analogy to the well-known effects of instrumental conditioning: Durations of rewarded percepts were longer than non-rewarded percepts, whereas punished percepts were shorter than non-punished percepts. Interestingly, conditioning effects seemed to depend, at least in the punishment experiment, on compensatory changes in the non-conditioned percept. Whereas punished percept durations remained largely unchanged, non-punished percepts increased significantly during punishment compared to baseline. This pattern is in line with a longstanding proposition in binocular rivalry research which states that increasing or decreasing the ‘strength’ of one stimulus during perceptual competition leads to a compensatory decrease or increase of perceptual dominance durations of the other stimulus whereas dominance durations of the manipulated stimulus stay the same (Levelt’s second proposition, Levelt, 1968; but see also Bossink et al., 1993; Brascamp et al., 2006). Even though the evidence for such a compensatory change in dominance durations was less clear in the reward experiment, one might conclude that this finding could be specific to binocular rivalry. Alternatively, the effects of conditioning on phase durations could be interpreted as a general increase of percept durations during conditioning and a relative decrease from this trend in the non-favorable percept (i.e., the non-rewarded or the punished percept). Generally increased percept durations during conditioning might be due to rivalry-specific long-term effects, e.g., related to diminishing interocular suppression over time (Hollins and Hudnell, 1980; Klink et al., 2010), or increased motivation to detect targets, which might have further lowered the attention to perceptual alternations and thereby increased percept durations (Paffen et al., 2006; Frassle et al., 2014). Lower attention to perceptual alternations might also explain the generally longer percept durations in our target detection paradigm. Importantly, however, the general pattern of opposing reward and punishment effects on perception will serve as a starting point for future research investigating whether other forms of perceptual inference are shaped by instrumental learning in a similar way.

In both experiments reported here, the effects of conditioning were no longer detectable as soon as reward or punishment had been stopped, which is compatible with extinction as observed in behavioral (Bouton, 2002) as well as emotional learning (Rescorla, 2000; Phelps et al., 2004). Extinction is thought to reflect active learning rather than a passive decay of previously learned associations (Bouton, 2002; Hartley and Phelps, 2010). The absence of prolonged conditioning effects in both our experiments might therefore point to a continuous adaptation of perceptual inference to changing conditions. It should be noted, however, that we did not observe a statistically significant reduction of the conditioning effect in the extinction phase, which precludes strong conclusions regarding extinction effects in our experiment. Future research might reveal whether effects of conditioning on perception quickly vanish (as e.g., observed with unconsciously acquired fear, Raio et al., 2012) or persistently shape future perception based on the learning history.

Given previous evidence for voluntary influences on multistable perception (Meng and Tong, 2004; Zhang et al., 2012) one might wonder whether participants in our study had realized which percept was more beneficial (i.e., the rewarded percept in Experiment 1 or the non-punished percept in Experiment 2) and tried to bias their perception accordingly. However, this interpretation can be discarded for a number of reasons. Most importantly, our assessment of perception relied on an indirect and objective measure and was based on a task that was orthogonal to the actual parameter of interest, that is, change in perceptual dominance duration as a function of conditioning. As a consequence, the majority of participants remained unaware of the subjective nature of perceptual alternations, rendering the possibility that they might have tried to voluntarily influence perception highly unlikely. Accordingly, only very few participants applied strategies that could have yielded differential effects on the two rivaling percepts. Crucially, even after rigorous exclusion of all participants in whom awareness of rivalry or any use of strategy could be suspected, the effects of conditioning remained stable. Moreover, eye tracking analyses indicated that the frequency of eye blinks, which can induce perceptual switches, was balanced between the two percepts and was thus unlikely to have influenced perceptual dominance durations in any systematic way. For these reasons, the observed effects on perceptual dominance durations seem likely to be due to an automatic adaptation of perceptual inference rather than being mediated by any voluntary strategy or even a reporting bias. Our results are thus also in line with findings of implicit, i.e., unconscious, effects of reward on behavior (Pessiglione et al., 2007) and now extended this evidence to the domain of perception. However, given the dependency of our measure on behavioral responses to targets for which we cannot calculate a response bias, we currently cannot completely exclude the possibility that conditioning affected response selection subliminally (cf. Pessiglione et al., 2008) and thus biased even our indirect measure of perception. Evidence from other independent measures of perception such as pupil diameter (Einhauser et al., 2008) or neural activation (Haynes and Rees, 2005; Schmack et al., 2013) may help to further substantiate the notion of conditioned perception by ruling out the possibility of subliminal conditioning effects on response selection with even higher certainty. With regard to the question of involvement of conscious processes, future studies should also account for the methodologically challenging assessment of contingency awareness (Lovibond and Shanks, 2002). Since participants in the present study showed a residual albeit low level of awareness for the contingency of reward and punishment, the present study cannot claim complete absence of contingency awareness, but nevertheless provides first evidence that perceptual inference is influenced by reinforcement independent of voluntary control.

Our results are in general agreement with studies that reported effects of current needs and preferences or stimulus value on perceptual inference (Proshansky and Murphy, 1942; Schafer and Murphy, 1943; Haggard and Rose, 1944; Bruner and Goodman, 1947; Balcetis and Dunning, 2006, 2010; Fleming et al., 2010; Summerfield and Koechlin, 2010; Anderson et al., 2011c; Balcetis et al., 2012; Radel and Clement-Guillotin, 2012; Schmack et al., 2013). However, our findings go substantially beyond this previous work in several ways. First, unlike prior studies we did not rely on participants’ report but used an orthogonal task to test our hypothesis with regard to changes in perception that was independent of participants’ intention and report bias (only Balcetis et al., 2012; Schmack et al., 2013, used some report-independent measures of perception). Second, we could demonstrate that perceptual inference is not only affected by the predefined value of a stimulus, but that it is shaped adaptively by its consequences. Third, conditioning exerted its effects on perception implicitly, that is, without observers even being aware of the possibility of such effects. Finally, we could show opposite effects of reward and punishment, in analogy to the effects of instrumental conditioning on action. The latter point is of particular interest when discussing our findings in relation to the earlier observation that stimuli previously paired with reward are more difficult to ignore in an attentional selection task (Anderson et al., 2011a). It has been suggested that pairing of stimuli with punishment would also render them more salient and therefore similarly difficult to ignore (Anderson, 2013). In fact, visual stimuli that have been paired with an aversive event or negative gossip tend to dominate perception during binocular rivalry, i.e., are more likely to be selected by perceptual inference (Alpers et al., 2005; Anderson et al., 2011c). In contrast, pairing the outcome of perceptual inference with an aversive consequence in our study (Experiment 2) yielded the opposite effect with predominance of the non-punished percept over the punished one. A crucial difference between the present and earlier studies regards the precise subject of associative pairing: In previous studies (Alpers et al., 2005; Anderson et al., 2011c), negative values were associated with a *stimulus*, whereas in our present study values were associated with a *percept*, that is, the outcome of perceptual inference. A possible conclusion is that changing the value of a stimulus, no matter whether positive of negative, increases its affective salience, which in turn facilitates its access to awareness. In contrast, when it is the outcome of perceptual inference that is associated with a value, the effect depends on the sign of this value: a positive value will increase the probability of the associated perceptual outcome, while a negative value decreases its probability. To the best of our knowledge, our study is the first to directly demonstrate such differential effects of conditioning with positive and negative consequences on perception.

In conclusion, our results suggest that the inferential processes that give rise to our conscious perceptual experience are subject to the same well-established effects that instrumental learning is known to have on action selection. This finding narrows the gap between the conceptually separated domains of perception and action and is therefore of fundamental importance for the understanding of the adaptive brain mechanisms underlying the perception of and the interaction with the environment. It will be an intriguing challenge for future research to elucidate the neural mechanisms involved in the shaping of perception by instrumental learning.

## Conflict of Interest Statement

The authors declare that the research was conducted in the absence of any commercial or financial relationships that could be construed as a potential conflict of interest.
