# Supplementary material for: Reinforcement of perceptual inference: reward and punishment alter conscious visual perception during binocular rivalry
Source: Front Psychol. 2014 Dec 3;5:1377. doi: 10.3389/fpsyg.2014.01377 (PMC4253824; doi:10.3389/fpsyg.2014.01377)
Supplement: Supplementary file 1 [file Data_Sheet_1.DOCX]

***Supplementary Material***

**Reinforcement of perceptual inference: reward and punishment alter conscious visual perception during binocular rivalry**

**Gregor Wilbertz*, Joanne van Slooten, Philipp Sterzer**

Visual Perception Lab, Department of Psychiatry and Psychotherapy, Charité – Universitätsmedizin Berlin, Germany

*** Correspondence:** Gregor Wilbertz, Department of Psychiatry and Psychotherapy, Charité – Universitätsmedizin Berlin, Charitéplatz 1, 10117 Berlin, Germany. Email: gregor.wilbertz@charite.de

1. **Supplementary Results**

**1.1 Experiment 1**


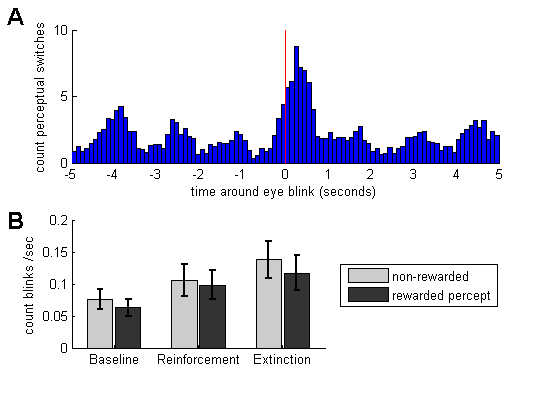
**Figure S1.** **Eye blink analysis of Experiment 1.** During binocular rivalry, probability for a perceptual switch was increased immediately after an eye blink (as indicated by more percept ends after a blink) (A). The frequency of eye blinks increased over the experiment but did not differ between the two percepts at baseline or during reinforcement (all *p*s>.20) (B). Note: Eye blink data were available only for a subset of N=18 participants. Error bars denote standard errors of the mean.

Contingency awareness: Awareness of the aim of the study was generally low. Only 5 out of 37 participants (13.5%) noticed the subjective nature of perceptual changes (e.g., ‘It seemed to depend on my eyes.’). They were therefore excluded from the conservative subsample analysis. Of the remaining participants, 30 (81.1%) did not notice any subjective influences on perception and 2 (5.4%) were unsure. Only 1 out of 37 participants (2.7%) reported to have used a strategy which might have selectively affected durations of one but not the other percept (‘increased concentration during the perception of red’) and was thus also excluded from the conservative subsample analysis. Four additional participants (10.8%) mentioned the use of strategies that potentially could have affected perceptual dominance durations in general (e.g. ’more eye blinks’ or ‘more button presses’), but were not directly related to a particular color or eye; they were therefore not excluded from the conservative subsample. The remaining 32 participants (86.5%) explicitly stated no use of any potentially confounding strategy during the experiment. With regard to reinforcement contingency awareness, only 3 out of 36 participants^[[1]](#footnote-1)^ (8.3%) correctly identify the rewarded color when asked for any relationship with reward delivery. Asking cued questions (i.e. whether participants noticed any relationship between reward and color) revealed that only 8 out of 35 participants^[[2]](#footnote-2)^ (22.9%) could be regarded as ‘contingency aware’ as well as additional 10 participants (18 in total, 51.4%) at least temporarily thought about a possible association between color and reward but finally decided to reject this hypothesis. In the 2AFC (red or blue?) question that was given to a subset of 29 participants^[[3]](#footnote-3)^, 19 (65.5%) correctly guessed the rewarded color (binomial test: *p*=.068).

Subsample analysis: As already indicated in the main text, analysis of the remaining 15 participants after exclusion due to any potentially confounding factor revealed almost the same results as for the whole sample. Baseline comparisons of dominance durations yielded no differences between percepts either with regard to eye (left-eye stimulus *M*=7.81, SD=4.42, right-eye stimulus *M*=7.10, SD=3.40, *t*[14]=1.19, *p*=.254), color (red stimulus *M*=7.21, SD=3.40, blue stimulus *M*=7.69, SD=4.43, *t*[14]=0.78, *p*=.449), or reward (later-on rewarded percept M=7.23, SD=3.23, later on non-rewarded percept *M*=7.68, SD=4.56, *t*[14]=0.72, *p*=.482). Among participants in the subsample 7 out of 12 (58.3%) correctly guessed the rewarded color in the 2AFC question (binomial test: *p*=.194).

**1.2 Experiment 2**

Contingency awareness: Detailed debriefing of participants revealed similar results as in the first experiment. Only 6 out of 34 participants (17.7%) reported to have noticed some endogenous influences on perceived color or color change and were therefore excluded from the conservative subsample analysis. Two additional participants (5.9%) said they were not sure. The remaining 26 participants (76.5%) clearly said they believed color changes were displayed on the computer screen. With regard to voluntary influences of perceptual durations, 4 out of 34 participants (11.9%) reported to have used a strategy which might have selectively affected durations of one but not the other percept and were therefore excluded from the conservative subsample analysis. An additional 5 participants (14.7%) mentioned the use of strategies that could have potentially affected perceptual dominance durations in general, but were not systematically related to a particular color or eye. They were therefore not excluded from the conservative subsample analysis. The remaining 25 participants (73.5%) explicitly stated no use of any potentially confounding strategy during the experiment. With regard to punishment contingency awareness, only 3 out of 34 participants (8.8%) correctly identified the punished color when asked about any relationship with punishment delivery. Two additional participants (5.9%) assumed that punishment might have been related to color but could not decide to which one. Asking cued questions (i.e., whether participants noticed any relationship between monetary loss and color) revealed only 11 out of 34 participants (32.4%) who could be regarded as ‘contingency aware’ as well as additional 2 participants (13 in total, 38.24%) who at least temporarily thought about a possible association between color and monetary loss but finally decided to reject this hypothesis. Lastly, 23 out of 34 participants (67.7%) correctly guessed the punished color in a 2AFC (red or blue?) questions (binomial test: *p*=.023).


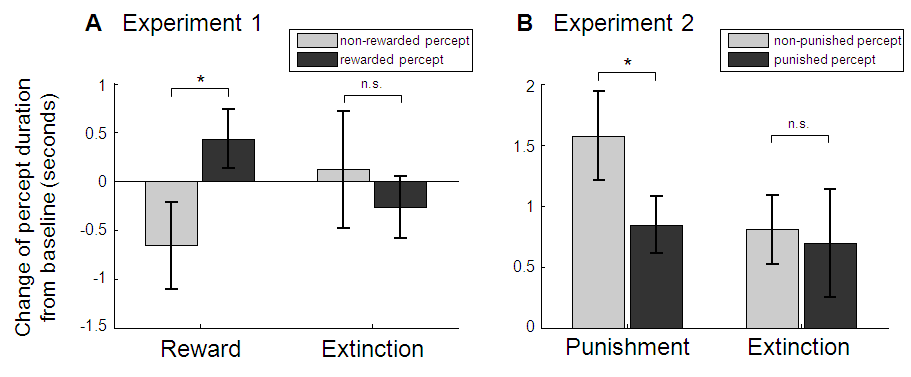


**Figure S2.** **Results of the conservative subsample analyses after exclusion of participants.** As in the whole sample (see main text), baseline corrected rewarded percepts were significantly longer than non-rewarded percepts during the conditioning blocks in Experiment 1 (A). Punished percepts during the conditioning blocks in Experiment 2 were significantly shorter than non-punished percepts compared to baseline (B). Reward and punishment effects were not persistent as indicated by no significant differences (n.s.) between percept durations during the extinction blocks. Note: sample size was *n*=15 and *n*=15 for Experiment 1 and 2, respectively, for these subsample analyses only; * *p*<.05, paired t-test. Error bars denote within-subject standard errors.

Subsample analysis: Results for the remaining 15 participants in the conservative subsample were almost the same as for the whole group. Baseline comparisons of percept durations yielded no differences with regard to eye (left-eye stimulus *M*=5.67, SD=1.79, right-eye stimulus *M*=6.06, SD=2.06, *t*[14]=1.53, *p*=.149), or color (red stimulus *M*=6.03, SD=1.83, blue stimulus *M*=5.70, SD=2.03, *t*[14]=1.26, *p*=.228), but a trendwise significant difference between the later-on punished percept (*M*=6.08, SD=2.00), and later-on non-punished percept (*M*=5.64, SD=1.85, *t*[14]=1.78, *p*=.097). Among participants in the subsample 11 out of 15 (73.3%) correctly guessed the punished color in the 2AFC question (binomial test: *p*=.059).


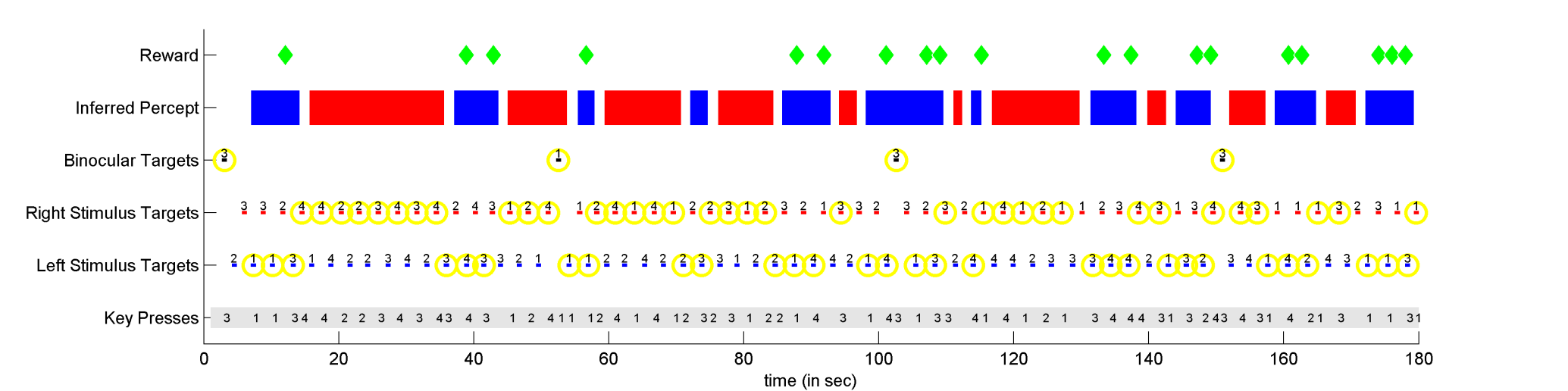


**Figure S3. Original data of an exemplary block of target detection performance and indirectly inferred percept during binocular rivalry.** The lower row depicts key presses with the numbers representing keys 1-4 (for up, down, left and right) and the position on the x-axis representing the onset of the corresponding key press. Similar, numbers in the three rows in the middle represent locations of the corresponding targets within the stimulus (1-4 for up, down, left and right). Left stimulus targets were presented to the left eye only, right stimulus targets to the right eye only, and bilateral targets were presented simultaneously on both stimuli (at the same position). Yellow circles indicate a correct response to the corresponding target within the defined response window. The current percept was inferred indirectly based on hits and misses of targets (see text of the main manuscript for details on the percept inference algorithm). Note, only periods with a clear inference are considered as percepts, whereas ambiguous periods are left empty. Onsets of acoustically presented rewards are indicated by green diamonds in the top row of the graph.

1. Note: detailed debriefing data on this question was not available for one participant, who, however, denied using any behavioral strategy (see above). [↑](#footnote-ref-1)
2. Data are missing for 2 participants. Again, none of them had used a confounding strategy. [↑](#footnote-ref-2)
3. Data are missing for 8 participants. None of them had used a confounding strategy. [↑](#footnote-ref-3)
